# Parallel and cross-resistances of clinical yeast isolates determined by susceptibility pattern analysis

**DOI:** 10.3205/id000020

**Published:** 2016-06-07

**Authors:** Arno F. Schmalreck, Birgit Willinger, Evgeny A. Idelevich, Christian Fegeler, Cornelia Lass-Flörl, Wolfgang Fegeler, Karsten Becker

**Affiliations:** 1 Mikrobiologische Beratung und Service (MBS), München, Germany; 2 Division of Clinical Microbiology, Department of Laboratory Medicine, University Vienna, Austria; 3 Institute of Medical Microbiology, University Hospital Münster, Germany; 4 Medical Informatics, Faculty of Informatics, University Heilbronn, Germany; 5 Section Hygiene and Medical Microbiology, Medical University Innsbruck, Austria

**Keywords:** Candida, antifungals, flucytosine, amphotericin B, fluconazole, voriconazole, posaconazole, caspofungin, micafungin, anidulafungin, susceptibility testing, susceptibility pattern analysis, parallel resistance, cross-resistance

## Abstract

For calculated initial antifungal therapy, knowledge on parallel and cross-resistances are vitally important particularly in the case of multiresistant isolates. Based on a strain collection of 1,062 yeast isolates from a German/Austrian multicentre study, susceptibility pattern analysis (SPA) was used to determine the proportion of parallel and cross-resistances to eight antifungal agents (AFAs) encompassing flucytosine, amphotericin B, azoles (fluconazole, voriconazole and posaconazole) and echinocandins (caspofungin, micafungin and anidulafungin). A total of 414 (39.0%) isolates were resistant for one or more of the AFAs. Resistance to one AFA was shown for 18.1% of all isolates. For 222 isolates (20.9%), resistance to two to seven AFAs was noted (7.7%; 7.7%; 3.6%; 1.0%; 0.7% and 0.2% to 2, 3, 4, 5, 6 and 7 antifungal compounds, respectively). Partial parallel resistances within the azole and echinocandin classes, respectively, were found for 81 (7.6%) and 70 (6.6%) isolates. Complete parallel resistances for azoles, echinocandins and combined for both classes were exhibited by 93 (8.8%), 18 (1.7%) and 6 (0.6%) isolates, respectively. Isolates displaying cross-resistances between azoles and echinocandins were infrequently found. Highly resistant isolates (resistance to ≥6 AFAs) were almost exclusively represented by *Candida albicans*. Highly standardized testing of AFAs in parallel and from the same inocula followed by SPA allows detailed insights in the prevalence and distribution of susceptibility patterns of microbial isolates.

## Background

Treatment options for invasive fungal infections are restricted by a very limited number of applicable antifungal agent (AFA) classes. Due to extensive worldwide use of fluconazole in the past decades, azole resistance has been significantly emerged, often associated with clinical failure [1], [2], [3], [4]. Also for the other AFA classes, resistances with substantial consequences for treatment and patient outcome have been increasingly reported [5], [6], [7], [8], [9], [10]. 

There is mounting evidence that the serious phenomenon of multi-resistance has reached also infections due to fungal pathogens. While reports on testing susceptibilities of yeast isolates to individual antifungals are available for many parts of the world [11], [12], [13], [14], systematic data on parallel and cross-resistances of *Candida* and other yeast isolates towards azoles, echinocandins, polyenes and flucytosine are still rare [15]. Of note, the terms “parallel resistances” and “cross-resistances” are often undifferentiated and/or varyingly used today. Here, parallel resistance (PR) was defined as resistance of a given isolate to all antifungal agents within an antifungal class and cross-resistance (CR) as resistance of a given isolate to antifungal agents belonging to different classes of antifungals.

Previously, we have analyzed the AFA susceptibilities of 1,062 yeast isolates recovered from clinical specimens within a collaborative study including 17 participating medical centres mainly by standard susceptibility testing analyses [12]. Here, susceptibility pattern (SP) analysis (SPA) was applied allowing a highly standardized analysis and true comparison of antifungal susceptibilities based on determined individual SP of each single isolate [16]. Based on this, we determined the proportion of parallel, cross- and multi-resistances for the clinically most prevalent *Candida* species, but also for rare yeast species of interest, isolate- and species-specifically stratified for azoles, echinocandins, flucytosine and amphotericin B.

## Methods

### Yeast isolates

A total of 1,062 clinical yeast isolates (species distribution, see Table 1 (Tab. 1)) were recovered from clinical relevant routine samples of hospitalized patients and tested for susceptibility within a German/Austrian collaborative study comprising 17 study centres [12]. Details regarding species-specific resistance profiles and AFA-related resistance prevalences have been previously reported [12]. Briefly, 184 (17.3%) specimens were recovered from blood and other normally sterile sites and 878 (82.7%) specimens comprised those from non-sterile sites, such as specimens from lower respiratory tract (n=299; 34.1%), mouth and throat (n=126; 14.4%), urinary tract (n=166; 18.9%); female genital tract (n=35; 4.0%), gastro-intestinal tract (n=61; 6.9%)] and other sites (n=191; 21.7%).

### AFA and susceptibility testing

Susceptibility testing was performed as previously published according to DIN (Deutsches Institut für Normung e.V., *i.e.* the German Institute for Standardization) [12]. Briefly, antifungal agents were tested in parallel in yeast sensitivity test (YST) as log_2_-dilution-rows in ready-to-use microdilution trays manufactured by Merlin GmbH (Bornheim-Hersel, Germany) comprising the following range of antifungal concentrations (in mg l^-1^): amphotericin B (AMB), 0.008–8.0; flucytosine (FCY), 0.31–32.0; fluconazole (FLC), 0.063–64.0; posaconazole (POS), voriconazole (VOR), anidulafungin (ANI), caspofungin (CAS), and micafungin (MCA), 0.008–8.0. YST medium, which corresponds to the modified HR medium according to DIN 58940-8415 [17], was manufactured by Sifin GmbH (Berlin, Germany). Endpoint reading was performed at 24 h and the MICs were controlled after 48 h incubation at 35°–37°C. Minimal inhibitory concentrations (MICs) were determined according to German DIN standards, *i.e.* the assumed endpoint was the lowest concentration which shows agitated a significant less turbidity than those of 80%-inhibitions control by visual inspection [18], [19].

Statistical analysis was performed with SAS^®^ software (SAS Institute, Cary, NC, USA). The antilog of the calculations was displayed as MICs from calculated results. Differences were assessed by using Chi squared test; P values lower than 0.05 were considered statistically significant.

### MICs and MIC interpretive criteria

Details of the *in vitro* susceptibilities of yeast isolates collected within the multicenter study towards eight antifungal agents have been reported before [12]. Briefly, all drug classes demonstrated multi-modular, at least bi-modular MIC distributions. The geometric means of MICs for FLC, POS, VOR, ANI, CAS, MCA, AMB, and FCY for *Candida albicans* strains (n=573; mg l^-1^) were 0.6, 0.5, 2.0, 0.1, 0.1, 0.03, 0.1 and 0.03 and for non-*C. albicans* strains (n=473; mg l^-1^): 0.8, 0.9, 8.2, 1.3, 0.4, 0.4, 0.2 and 0.04, respectively.

MIC categorisation was performed as published by applying interpretative criteria for AMB as published by EUCAST [20] and for FLC as published by DIN [17]. For POS, ANI, CAS, and MCA interpretative criteria published by Pfaller et al. were used [21], [22]. CLSI criteria were applied for FCY and VOR [23]. Interpretative criteria for susceptible (S) and resistant (R) were: AMB: S≤1.0, R>1.0; FCY, FLC: S≤4.0, R>16.0; POS, VOR: S≤1.0, R>2; and ANI, CAS MCA: S≤2, R>2.0. If appropriate, the values in between were used as intermediate (I). Species-specific breakpoints were not applied.

SPs representing sequences of interpretative categories (S, I or R) in a prefixed order of the test results were determined by SPA as described, here in an adaptation for fungal microorganisms [16]. An SP as applied here contains the number of members of antifungal class and the S-I-R categorization (*e.g.* for a given isolate with complete azole resistance to FLC, POS and VOR: 3R=R-R-R or for an isolate tested susceptible to all eight AFAs included: 8S=S-S-S-S-S-S-S-S).

### Resistance definitions

Definitions were applied according to DIN 58940-1 and as published elsewhere [12], [17]. Briefly, multiple resistance (MR) was defined in this study, when two or more antifungal agents independently of any substance class were tested resistant in the same isolate,* i.e.* representing a random susceptibility pattern. Parallel resistance (PR) was defined as resistance of a given isolate to all (complete PR) or more than one, but not all (partial PR) AFAs within a class of antifungals. Cross-resistance (CR) was defined as resistance of a given isolate to two or more AFAs belonging to different classes of antifungals. 

## Results

A total of 1,062 clinical yeast isolates (*C. albicans*, n=573; 54.0%; non-*albicans Candida* spp., n=473; 44.5%; other yeasts, n=16; 1.5%; further details in Table 1 (Tab. 1)) were enrolled for SPA evaluating their parallel and cross-resistance patterns. Unless otherwise stated, all results given in the text and the tables are based on the application of YST medium and endpoint reading after 48 h.

For FCY, FLC, VOR and POS, a threefold categorization (S, I and R) was used and a twofold categorization (S and R) was applied for AMB, CAS, MCA and ANI. Thus, the individual SP of a given isolate represents one SP out of a variety of 1,296 theoretically possible SPs. However, only a limited amount of SPs were found, nevertheless, demonstrating numerous parallel and/or cross-resistant strains. Consequently, only a selection of relevant data restricted (i) to clinically most prevalent *Candida* species or (ii) to rare yeast species with noticeable SPs has been included in Table 1 (Tab. 1), Table 2 (Tab. 2), Table 3 (Tab. 3), Table 4 (Tab. 4), Table 5 (Tab. 5), Table 6 (Tab. 6) and Table 7 (Tab. 7). Overall, 62 SPs and 117 SPs were found after 24 h and after 48 h incubation using YST medium. In contrast, applying RPMI medium, the amount of SPs gained after 24 h (n=54) as well as after 48 h (n=86) incubation were less than applying YST medium. However, only the medium-caused difference of SP numbers observed after 48 h reached significance (p<0.05) (data not shown).

Standard susceptibility testing analysis neglecting the individual patterns of susceptibility was compared with SPAs considering isolate-specifically resistances within AFA classes and in between different AFA classes (Table 2 (Tab. 2)), thus, allowing inferences to all naturally occurring SPs in detail. Similar analyses are given for echinocandins and, where appropriate, for FCY and AMB (Table 2 (Tab. 2)). Considering isolates exhibiting exclusive resistance to only one of the AFAs tested, but otherwise tested susceptible (1R7S SP), 93 isolates were found to meet this condition. Including also those isolates otherwise tested susceptible or intermediate (1R7S/I SP), this condition was fulfilled by 192 isolates. While 113 (10.6%) isolates were found to be VOR-resistant by standard analysis, SPA demonstrated that none of these isolates showed exclusive VOR resistance (1R SP) analyzing those isolates tested resistant towards the three azoles included. In contrast, 21 (2.0%) and 23 (2.2%) isolates, respectively, exhibited sole resistance towards FLC and POS, but were tested susceptible towards the other azole agents (Table 2 (Tab. 2)). Considering all AFAs tested, only five (0.5%) isolates were characterized by exclusive resistance to VOR (Table 2 (Tab. 2)).

Overall, this strain collection comprised 519 (48.9%) yeast isolates tested susceptible to all AFAs included. Regarding azoles and echinocandins, respectively, entire susceptibility were noted for 619 isolates (58.3%) and 974 isolates (91.7%). For the most prevalent species, this 3S SP varied for azoles and echinocandins, respectively, as follows: *C. albicans*, 77.5% and 91.8%; *Candida glabrata*, 23.5% and 97.9%; *Candida krusei*, 4.3% and 100%; *Candida parapsilosis*, 67.2% and 73.4% and *Candida tropicalis*, 45.9% and 96.7%.

Partial parallel resistances (SPs: RSS, RRS, SSR, SRR, SRS and RSR; unconsidering further SPs containing “I” categorization) within the azole and echinocandin classes, respectively, were found for 81 (7.6%) and 70 (6.6%) isolates.

The percentage of complete parallel resistance to all azoles (3R) was 8.8%, while it was 1.7% to all echinocandins (Table 1 (Tab. 1), Table 3 (Tab. 3), Table 4 (Tab. 4)). A complete resistance towards all azoles combined with cross-resistance either to FCY or AMB occurred in 1.7 % or 2.2% of the isolates. For echinocandins, this 3R SP together with cross-resistance to FCY and AMB, respectively, was 0.1% and 0.4% (Table 1 (Tab. 1)). 

The proportion of complete parallel resistance varied species-specifically. Of particular interest, complete parallel echinocandin resistance was observed for 23.8% of all *Candida dubliniensis* isolates (Table 1 (Tab. 1)). A respective echinocandin 3R SP was also documented for *Candida sake* (50%) and both *Geotrichum* species included, however, here, a very limited number of isolates enrolled should be taken in consideration. In contrast, *C. albicans* and *C. tropicalis* exhibited this 3R-SP for less than 2% of the isolates. However, within the azole class, 3.1%, 6.1%, 12.5%, 14.1%, 14.8%, 15.4%, 23.7 and 50.0% of the *C. parapsilosis*, *C. albicans*, *Candida guilliermondii* (only n=8), *C. glabrata*, *C. tropicalis*, *Saccharomyces cerevisiae*, *C. krusei* and *Candida melibiosica* (only n=2) isolates, respectively, showed a complete parallel resistance. Of interest, 1.8% of the *C. albicans* isolates showing complete parallel azole resistance exhibited also AMB resistance, while only 0.4% of complete echinocandin-resistant isolates of this species were tested also AMB-resistant. Other cross-resistance patterns towards AMB and FCY for complete azole- and echinocandin-resistant isolates, respectively, are given in Table 1 (Tab. 1).

A more detailed overview of cross-resistances for those yeasts exhibiting a complete parallel resistance pattern to all azoles (3R; 8.8%) and echinocandins (3R; 1.7%), respectively, is given in Table 3 (Tab. 3) and Table 4 (Tab. 4). Overall, 19.4 and 24.7% of complete azole-resistant yeast isolates were tested resistant also towards FCY and AMB, respectively. Regarding echinocandins, 7.5%, 12.9% and 14.0% of these isolates showed resistance to MCA, CAS and ANI, respectively. Of interest, while all full azole-resistant *C. glabrata* and *C. krusei* isolates were still susceptible towards all echinocandins, 31.4%, 28.6% and 20.0% of full azole-resistant *C. albicans* isolates showed cross resistance to CAS, ANI and MCA (Table 3 (Tab. 3)).

A complete parallel resistance to echinocandins was observed for only 18 yeast isolates. Of these, 16/1046 (1.5%) were represented by species of the *Candida genus*. Also both *Geotrichum* isolates included exhibited this SP (Table 4 (Tab. 4)). Overall, 5.6% and 22.2% of complete echinocandin-resistant yeast isolates were tested resistant also towards FCY and AMB, respectively. Noteworthy, 33.3%, 38.9% and 44.4% of these isolates were also categorized as resistant to VOR, FLC and POS, respectively. For complete echinocandin-resistant *C. albicans* isolates (n=10/57; 1.7%), 60% were tested resistant also towards each of the azoles included. All *C. dubliniensis* isolates of this category (n=5) were susceptible to VOR, while one isolate demonstrated partial parallel resistance to FLC and POS (Table 4 (Tab. 4)).

SPA results for rare yeast species, defined in this study as *Candida* and non-*Candida* yeast species comprising equal or less than five isolates, are given in Table 5 (Tab. 5). AMB-resistant isolates (each one isolate) were found for *Candida lipolytica*, *C. melibiosica*, *C. sake* and *Geotrichum* *candidum*. One *C. melibiosica* isolate showed complete parallel resistance to all azoles, but was susceptible to AMB, FCY and all echinocandins. The two *Candida norvegensis* isolates were tested resistant and intermediate, respectively, to FLC, but showed each a one-step more susceptible categorization towards VOR and POS. Both *C. sake* isolates offered resistances to echinocandins, one with complete parallel resistance and one remaining susceptible to MCA. A full echinocandin resistance was also noted for the *G. candidum* and *Geotrichum capitatum* isolates. The *Kodamaea ohmeri* isolate was tested susceptible to all AFAs with the exception of FLC (tested intermediate). 

**Figure 1:**
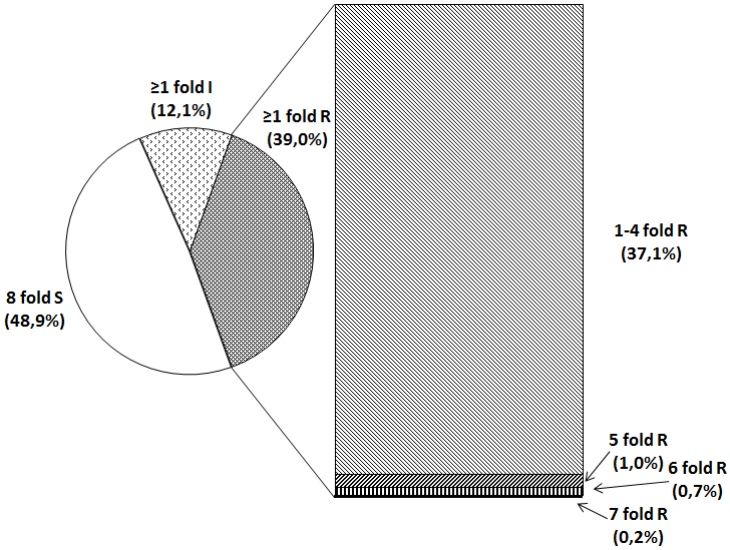
Proportion of major susceptibility patterns of study isolates (n=1,062) showing percentages of isolates (i) tested susceptible to all AFAs included (8S), (ii) non-resistant isolates tested intermediate to one or more AFAs (≥1I) or (iii) tested resistant to one or more AFAs (≥1R). For isolates exhibiting ≥1R SP, the percentages of multi-resistant isolates are given (1–4R, resistance to 1 to 4 AFAs; 5R, resistance to 5 AFAs; 6R, resistance to 6 AFAs and 7R, resistance to 7 AFAs).

Overall, complete susceptible SPs to all AFAs tested (8S-SP) were shown for 519/1062 isolates (48.9%) whereas 414 (39.0%) isolates were characterized by resistance to at least one of the AFAs included (≥1R) (Figure 1 (Fig. 1)). The remaining 129 isolates (12.1%) without resistances demonstrated in one or more cases intermediate susceptibilities distributed to seven different SPs.

Comparing the proportion of a species within the total amount of study isolates versus its proportion within those isolates exhibiting a complete susceptible phenotype (8S SP), *C. albicans* isolates showed significantly higher an 8S SP (54.0% vs. 75.9% 8S-SP; P<0.01), whereas respective isolates of *C. glabrata* (22.0% vs. 9.6% 8S-SP; P<0.01), *C. krusei* (4.3% vs. 0.2%; P<0.01) and *C. tropicalis* (5.7% vs. 2.5%; P<0.01) displayed significantly less this phenotype. 

For 122 (11.5% of total isolates) of the 129 intermediate-tested isolates comprising those without any result categorized as resistant, intermediate azole susceptibility was found as follows: FLC, n=43; POS, n=54; FLC and POS, n=20; FCY and FLC, n=3; FCY and POS, n=1; FCY, FLC and POS, n=1. Intermediate FCY susceptibility was noted for *C. albicans* (n=5) and *Candida kefyr* (n=2).

Overall, 222 (20.9%) isolates contained 81 SPs with more than one “R” within the pattern. No isolate was found to be resistant to all eight AFAs tested at 24 h and 48 h endpoint reading, respectively. Resistance limited to a single AFA was found in 192 (18.1%) isolates. In 222 isolates (20.9%), resistance patterns to 2–7 AFAs (2-7R) were noted. The distribution of isolates exhibiting resistance to ≥2 AFAs was as follows: 2R, n=82 (7.7%); 3R, n=82 (7.7%); 4R, n=38 (3.6%); 5R, n=11 (1.0%); 6R, n=7 (0.7%), and 7R, n=2 (0.2%). SPs with ≥5R reflecting pronounced multi-resistance are given in Table 6 (Tab. 6) and Figure 1 (Fig. 1). Of 140 isolates (13.2%) characterized by a 3–7-fold AFA resistance, 97 (9.1%) possessed a complete parallel resistance consisting of 79 (7.4%), 12 (1.1%) and 6 (0.6) isolates showing this SP against all azoles, echinocandins and both AFA classes, respectively. Of note, highly resistant isolates exhibiting 7R- and 6R-patterns were almost exclusively represented by *C. albicans* with one exception by *C. guilliermondii*. The two 7R *C. albicans* strains were still susceptible to flucytosine (Table 6 (Tab. 6)).

The proportion of still susceptible AFAs in relation to AFA-stratified resistances has been calculated in Table 7 (Tab. 7) for the clinically most relevant *Candida* species. As shown in Table 7 (Tab. 7), FLC-resistant *C. albicans* isolates (n=46) displayed susceptibility to one of the echinocandins in more than 76% (CAS, 76.1%; ANI, 78.3% and MCA, 84.8%), but only 13.0% of these FLC-resistant isolates were also susceptible to VOR. Noteworthy, for *C. glabrata*, this analysis demonstrated that almost all FLC- (n=55), VOR- (n=35) and POS- (n=104) resistant isolates were tested susceptible to all echinocandins with the exception of two CAS-resistant isolates.

While those *C. krusei* isolates tested resistant to FCY, AMB and/or one of the azoles revealed susceptibility to all echinocandins, respective *C. parapsilosis* isolates varied in the echinocandin susceptibility (Table 7 (Tab. 7)). Except one ANI-resistant *C. tropicalis* isolate, almost all isolates of this species exhibiting complete parallel resistance to the azole class were tested susceptible to all echinocandins included (Table 3 (Tab. 3) and Table 7 (Tab. 7)).

## Discussion

In addition to antibiotic resistance towards bacterial and other pathogens, nowadays, also resistance to AFAs has emerged as one of the international health challenges to be addressed. AFA-resistant phenotypes may develop in yeast populations due to mutations, selection processes and alternative mechanisms (e.g. biofilm formation) and *a priori*-resistant species and strains exist [24]. Moreover, recombination may play also a role in fungi [25]. The fundamental “answer” of the fungal pathogens to the selection pressure by an increasing use of AFAs, however, is represented by shifts in the species and strain distribution towards those species characterized by intrinsic resistances or increased capabilities to express resistance mechanisms [25]. While a shift toward infections caused by non-*albicans Candida* species have been globally reported [26], [27], [28], a systematic review by Falagas et al. covering the period between 1996 and 2009, showed significant geographic, study design and setting variations of the relative frequency of *Candida* spp. among cases of candidemia in different parts of the world, consequently, local epidemiological data continue to be of major significance [29].

Here, eight AFAs were tested in parallel, at the same time, in same assay, with the same inocula, thus, all assay-specific parameters were equal for all AFAs allowing a unique, highly standardized evaluation of the isolates’ susceptibilities. Considering pharmacological and pharmacodynamic aspects by a clinical breakpoint based categorisation (S-I-R), the results were arranged to individual SPs reflecting a resistance “fingerprint” for each single isolate, but embedded in the analysis of a large, recent multicentre isolate collection. Defining a fixed AFA sequence for SPA, SPs of different isolates can be easily compared and the frequencies of different SPs are determinable. Depending on the number of AFAs tested in parallel and the amount of parameters compared (*e.g.* methods, endpoint determinations, breakpoints, MIC-categorizations), a multitude of different SPs may have gained allowing detailed analyses of susceptibility distributions, for example, dependent on the methodical approaches used.

While standard descriptive methods and resulting data are the essential basis for questioning resistance preferentially for epidemiologically aspects, clinically and therapeutically relevant problems require comparative susceptibility evaluation methods. For this purpose, comparative AFA evaluation of individual isolate-specific susceptibilities may be useful, *e.g.* for determination of the prevalence of multi-resistant pathogens or to discover the susceptibility loss to complete AFA classes (*e.g.* azoles and echinocandins). For that purpose, SPA may act as useful tool allowing analyses of large strain collections down to the level of individual isolate-specific conclusions [30]. The data gained in this study by analyses of cross-susceptibility and -resistance patterns, respectively, are in particular relevant for treatment-related decisions. Here, of utmost clinical interest are those isolates exhibiting a complete parallel resistance to the entire azole class, in particular, if this is accompanied by a partial or, even worse, a complete echinocandin parallel resistance (Table 5 (Tab. 5) and Table 6 (Tab. 6)). In contrast to earlier presumptions that no complete echinocandin cross-resistance exists or that there would be only a low potential for the resistance development to echinocandins [8], [31], we could clearly demonstrate by SPA approach that complete parallel resistance within all echinocandins occurs, here found in 1.7% of the clinical routine isolates included. In comparison, the amount of complete parallel resistance within the azole class was 8.8% characterized by species-specific variations.

Although it is reported that clinical isolates with high echinocandin MICs tend to be low [32], isolates with echinocandin MICs of ≥4 mg/L were noted from 17 centres of this study comprising 88 isolates exhibiting those increased MICs for CAS (n=59; 5.6%), ANI (n=55; 5.2%) and MCA (n=26; 2.5%). Increased echinocandin MICs towards one, two and three AFAs of this class were displayed by 54/88 (61.4%), 16/88 (18.1%) and 18/88 (20.5%) of the isolates, respectively.

Complete echinocandin parallel resistance has been noted following prolonged use of these compounds for treatment of *C. albicans* and *C. parapsilosis* infections [33], [34]. Here, simultaneous presence of echinocandin- and species-dependent cross-resistance with azoles was found up to 30% depending on candidal species (Table 3 (Tab. 3) and Table 4 (Tab. 4)).

Selection pressure due to continuous exposure appears to play a crucial role in the emergence of azole resistance, thus, high parallel resistance rates for the azoles have to be noted as shown also in this study (8.8% of all isolates). This pattern is aggravated by cross-resistances of azole-resistant isolates to other AFA groups. In previous studies, none of 315 FLC-resistant *Candida* isolates demonstrated cross-resistance to ANI, whereas cross-resistance to CAS was rarely found (n=4; 1.1%) [35], [36]. In contrast, elevated cross-resistance frequencies of FLC-resistant yeast isolates (n=173) were found in this study for two echinocandins, ANI (n=18; 10.4%) and CAS (n=17; 9.8%). Cross-resistance between azoles and echinocandins, i.e. multi-resistance with different substance classes, may be caused by common resistance mechanisms such as over-expression of genes encoding efflux pumps, multi-drug transport systems, lipid-associated membrane (protein) functions and/or membrane fluidity [1], [37], [38], [39].

Complete azole-resistant yeast isolates of this study showed cross-resistance to AMB (n=23; 2.2%) and FCY (n=18; 1.7%). In contrast, cross-resistance of the echinocandin-resistant isolates to AMB (n=4; 0.4%) and FCY (n=1; 0.1%) was much rarer. As determined by SPA, a *C. sake* isolate showed parallel resistance to all echinocandins and cross-resistance simultaneously to AMB and FCY. Of note, this is the first report to our knowledge of those cross-resistance patterns.

Here, testing more than thousand clinical yeast isolates recovered within the course of a multicentre study to eight AFAs, a wide variety of SPs occurring was found. When tested highly standardized in parallel and from the same inoculum, evaluation of different AFA substances by SPA analysis allows detailed insights in the prevalence and distribution of susceptibility patterns of fungal isolates. Since the SPA approach enables a precise description of both known and so far unknown patterns of cross and parallel resistances, it may reflect the resistance situation in a given setting more comprehensively and more detailed compared to data based on standard susceptibility analyses. Consequently, deductions for treatment strategies based on species-specific SPs may be gained for improvement of calculated antifungal chemotherapy.

## Notes

### Acknowledgements

The authors would like to thank all members of the Antifungal Susceptibility Testing (AFST) Study Group involved in the German/Austrian multicenter study: F. Albert and C. Schoerner (Universitätsklinikum Erlangen, Institut für Klinische Mikrobiologie, Immunologie und Hygiene); O. Bader and M. Weig (Institut für Medizinische Mikrobiologie, Universitätsklinikum Göttingen); S. Crusius and A. Podbielski (Institut für Medizinische Mikrobiologie und Hygiene, Universität Rostock); V. Czaika (Dept. of Dermatology, Internal Medicine, Helios Kliniken, Bad Saarow); A. Haas (Institut für Medizinische Mikrobiologie der Ludwig-Maximilians-Universität München); G. Haase (Institut für Medizinische Mikrobiologie, RWTH Universitätsklinik, Aachen); M. Klotz and M. Herrmann (Institut für Mikrobiologie, Universitätsklinikum des Saarlandes, Homburg/Saar); K. Hochauf (Institut für Medizinische Mikrobiologie und Hygiene, Medizinische Fakultät, Technische Universität Dresden); H. Hof (Institut für Medizinische Mikrobiologie und Hygiene, Universitätsklinikum Mannheim); A. Rodloff (Institut für Medizinische Mikrobiologie und Infektionsepidemiologie, Universitätsklinikum Leipzig); M. Ruhnke (Medizinische Klinik und Poliklinik II, Onkologie und Hämatologie, Campus Charité Mitte, Humboldt-Universität, Berlin); U. Schumacher (Institut für Medizinische Mikrobiologie und Krankenhaushygiene, Universität Tübingen); L. Sedlacek and S. Suerbaum (Institut für Mikrobiologie und Krankenhaushygiene, Medizinische Hochschule Hannover); I. Sobottka (Institut für Medizinische Mikrobiologie, Virologie und Hygiene, Universitätsklinikum Hamburg-Eppendorf); G. Valenca and M. Abele-Horn (Institut für Hygiene und Mikrobiologie der Universität Würzburg).

The antifungal agents anidulafungin, fluconazole and voriconazole were provided free of charge by Pfizer GmbH (Germany) as were caspofungin by MSD (Germany), posaconazole by Essex (Germany) and miconazole by Astellas Pharma GmbH (Germany). This work was supported by a grant from Pfizer GmbH (Germany) for purchase and manufacturing of culture media, ready-to-use microdilution trays with the antifungal agents and transport material for collected strains. The opinions expressed in this article are those of the authors and do not necessarily represent those of the pharmaceutical companies.

### Conflict of interest

K.B. has received research support from Pfizer as well as lecture, travel and other fees from Cubist Pharmaceuticals, MSD Sharp & Dohme, Novartis Pharma and Pfizer. Ch.F. has received lecture, travel and other fees from Pfizer. B.W. has received research support from Pfizer as well as lecture, travel and other fees from Astellas Pharma GmbH, MSD Sharp & Dohme, and Pfizer. All other authors have no conflicts of interest to declare.

## Figures and Tables

**Table 1:**
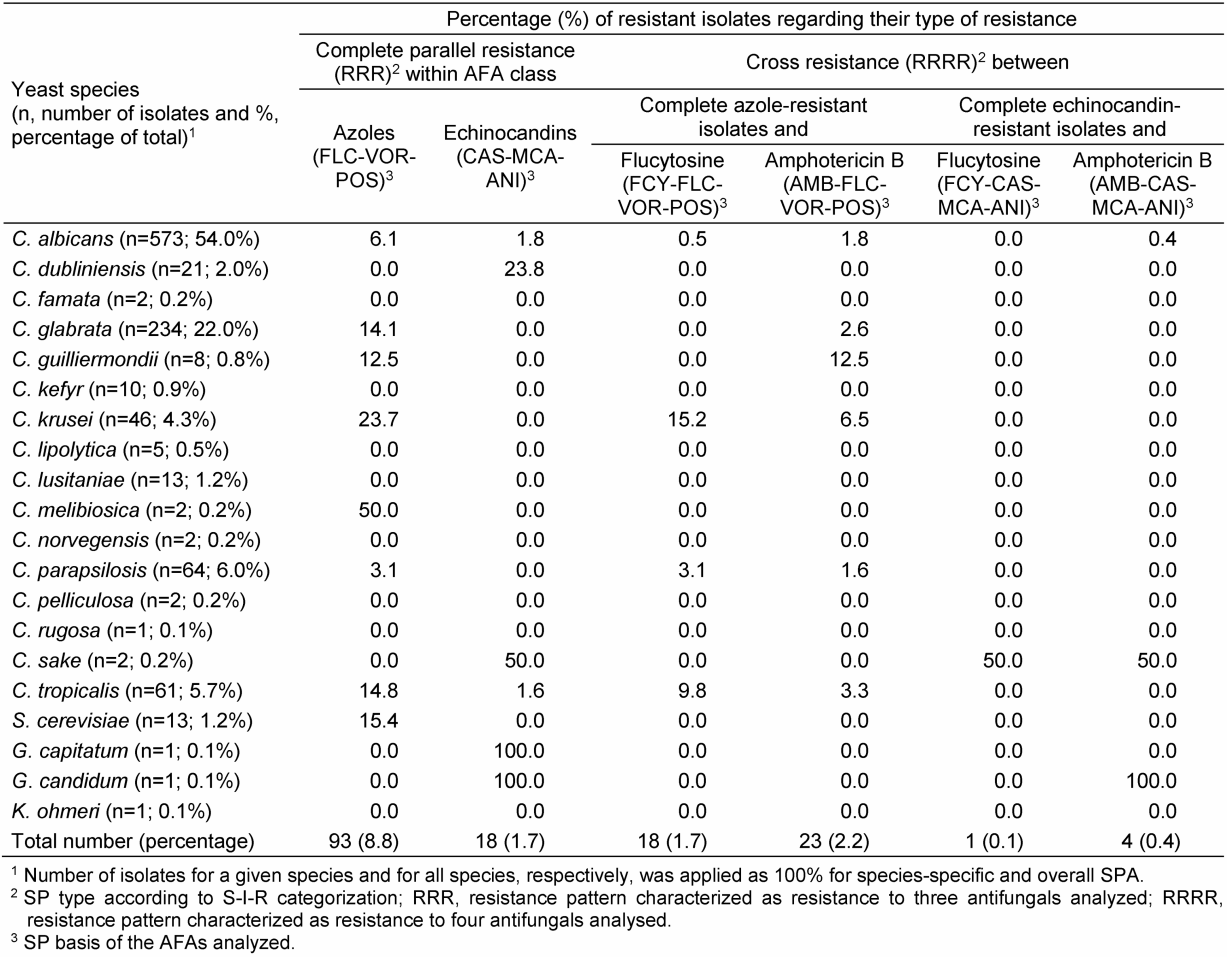
Percentages of yeast isolates (n=1,062) showing complete parallel resistance within azoles and echinocandins and their cross resistance patterns towards FCY and AMB

**Table 2:**
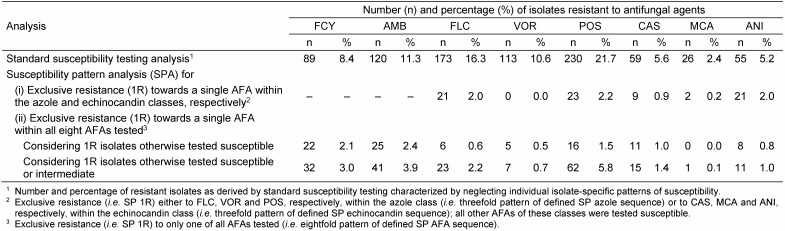
Standard analysis and SPA of susceptibility testing of yeast isolates (n=1,062)

**Table 3:**
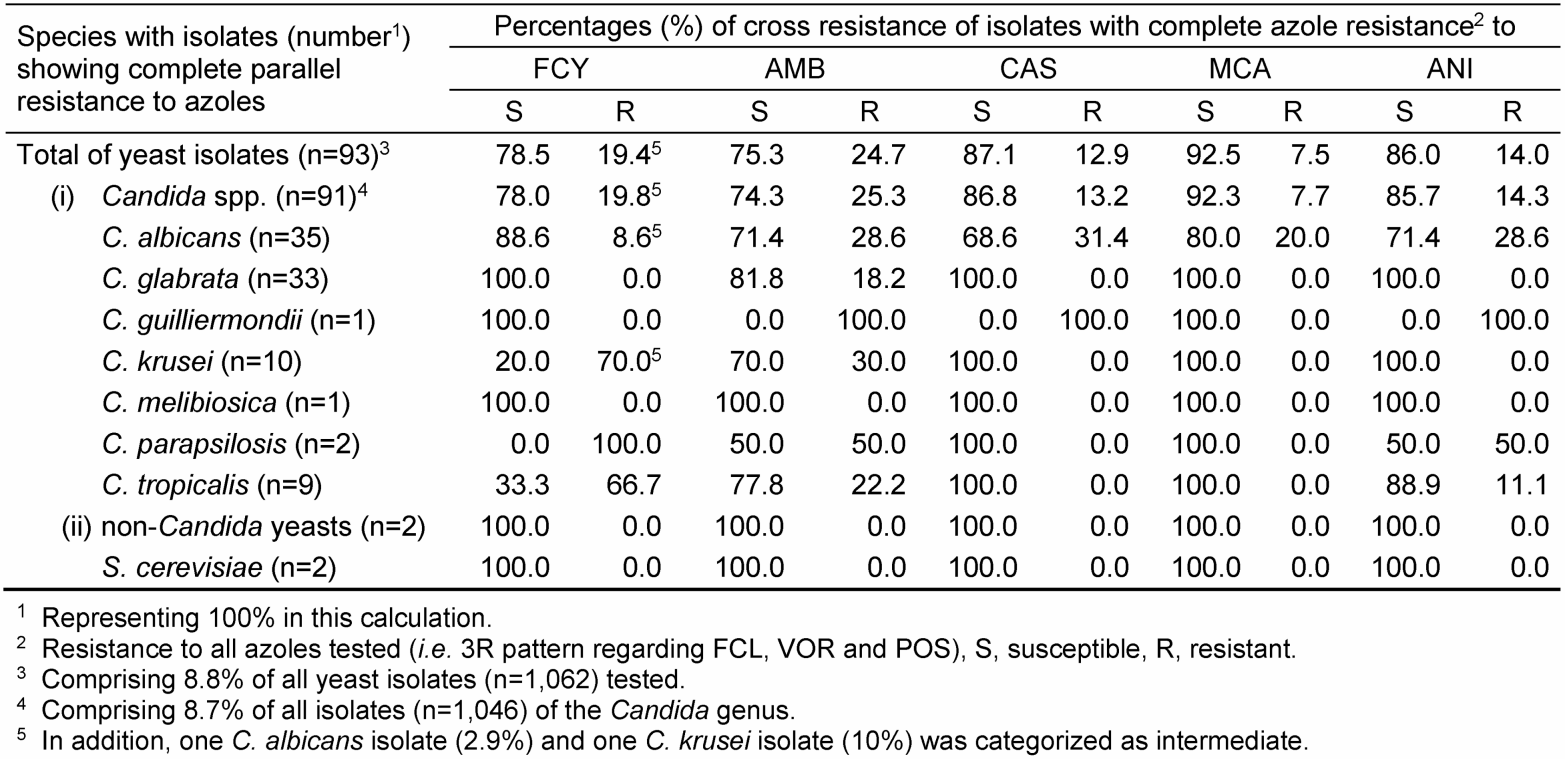
Proportion of cross resistances of isolates with complete parallel resistance within the azole class

**Table 4:**
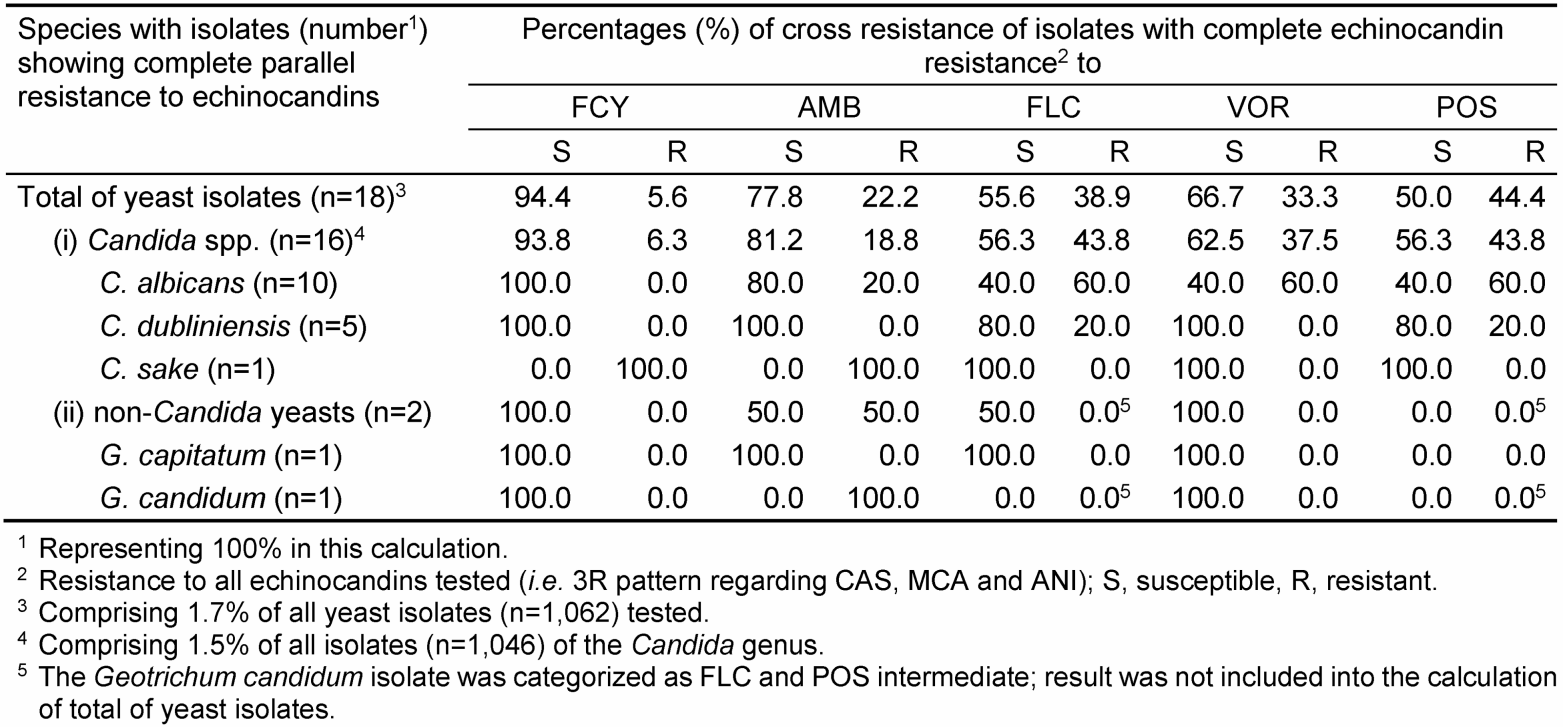
Proportion of cross resistances of isolates with complete parallel resistance within the echinocandin class

**Table 5:**
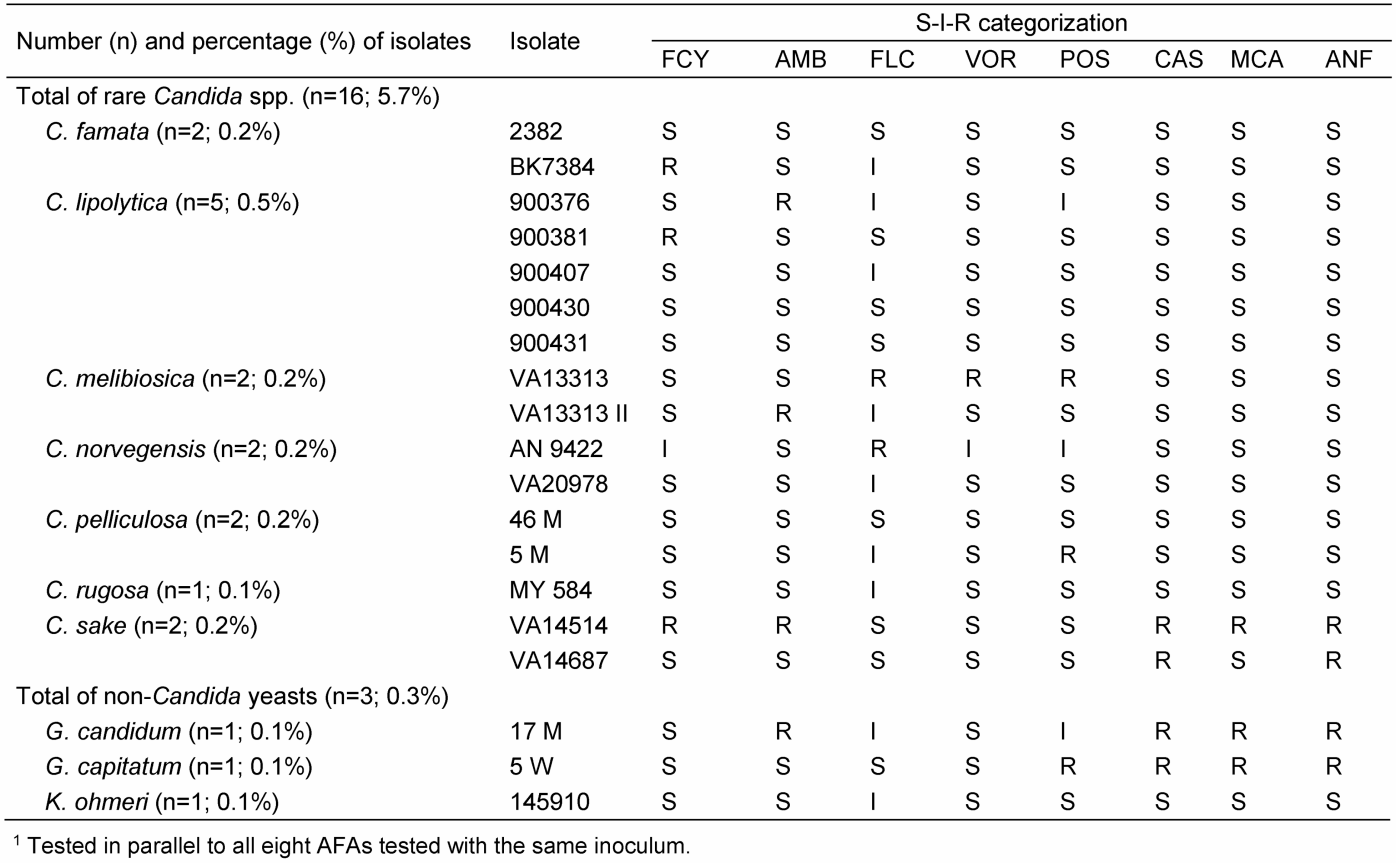
Detailed results of SPA for rare yeast species

**Table 6:**
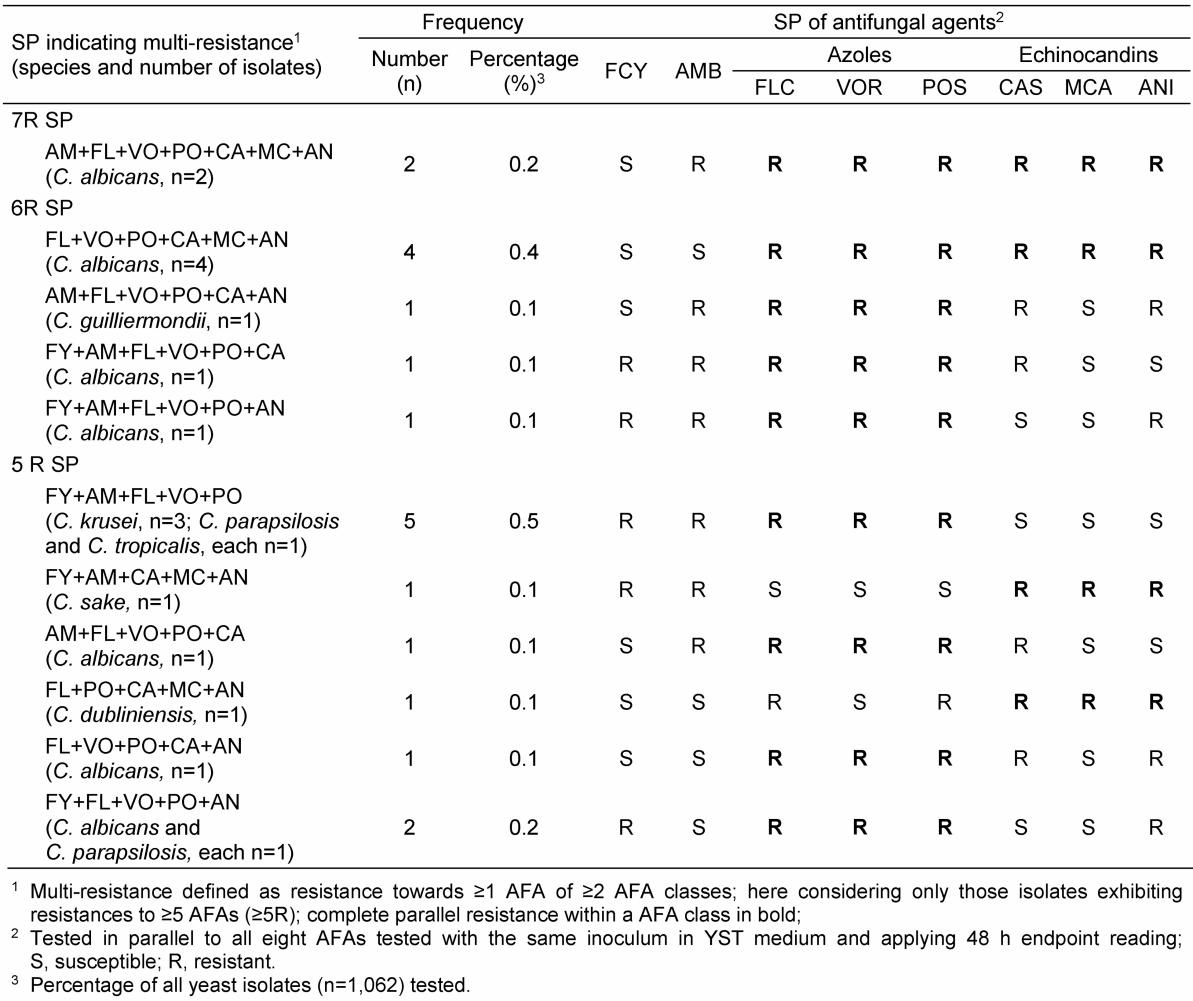
*Candida* isolates showing multi-resistant AFA patterns

**Table 7:**
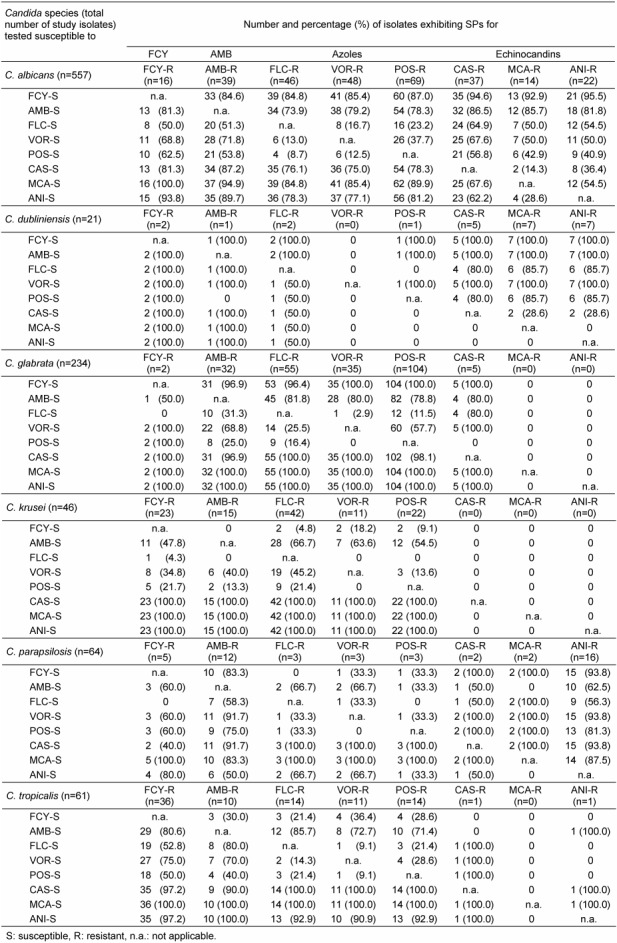
Proportion of still susceptible AFAs in relation to AFA-stratified resistances
